# Development and Experiment of Semi-Physical Simulation Platform for Space Manipulator

**DOI:** 10.3390/s24134354

**Published:** 2024-07-04

**Authors:** Jilong Xu, Yasheng Guo, Fucai Liu, Haoyu Huang

**Affiliations:** 1 Engineering Research Center of the Ministry of Education for Intelligent Control System and Intelligent Equipment, Yanshan University, Qinhuangdao 066004, China; xjl@stumail.ysu.edu.cn (J.X.); 15031527451@stumail.ysu.edu.cn (Y.G.); huanghaoyu@stumail.ysu.edu.cn (H.H.); 2 Key Lab of Industrial Computer Control Engineering of Hebei Province, Yanshan University, Qinhuangdao 066004, China

**Keywords:** space manipulator, semi-physical simulation, fractional-order linear self-adaptive disturbance rejection, motor driving force

## Abstract

To address the extended development cycle, high costs, and maintenance difficulties associated with existing microgravity simulation methods, this study has developed a semi-physical simulation platform for robotic arms tailored to different gravity environments and loading conditions. The platform represents difficult-to-model joints as physical objects, while the easily modeled components are simulated based on principles of similarity. In response to the strong coupling, nonlinearity, and excess force disturbance issues in the electric variable load loading system, a fractional-order linear active disturbance rejection control algorithm was employed. The controller parameters were tuned using an improved particle swarm algorithm with modified weight coefficients, and experimental results demonstrate that a fractional-order linear active disturbance rejection control improves response speed and disturbance rejection performance compared to linear sliding mode control. The study investigated the differences in the drive force of joint motors in space robotic arms under varying gravity environments and loading conditions. Experimental results indicate that load torque is the primary influencing factor on joint motor drive force, while radial force serves as a secondary influencing factor. Additionally, when the axis of the joint motor is perpendicular to the ground, it can, to some extent, simulate microgravity conditions on the ground.

## 1. Introduction

Joints, serving as pivotal components of robotic arms, play a direct role in determining the kinematics, dynamics, and control performance of the robotic arm. A typical joint is composed of various structures, including motors, reducers, encoders, and brakes, resulting in an exceptionally complex transmission system. Particularly in harmonic gear reducers, the flexspline undergoes deformation under varying loads, leading to uncertainty in model parameters and posing challenges to the establishment of accurate joint dynamic models using theoretical methods [1]. Moreover, the disparities in dynamics between gravitational and microgravity environments for space robotic arms necessitate substantial investments in funding, manpower, and facilities for conducting full-scale physical experiments using suspension, air flotation, or water flotation methods [2].

With the rapid advances in science and technology, semi-physical simulation technology has emerged as a potent tool for system development, offering advantages such as enhanced system development quality, shortened development cycles, and reduced development costs [3]. Consequently, the development of a semi-physical simulation device for robotic arms, the establishment of a semi-physical simulation system for robotic arms, simulation of the variation in gravitational and microgravity loads, differential analysis of joint drive forces, and related control research hold significant importance for enhancing the overall motion performance of robotic arms in operational environments.

Semi-physical simulation involves substituting challenging models with physical components and establishing a real-time loop between simulation and physical components to form a semi-physical simulation system. Initially, this method was predominantly applied in the development and testing of military equipment, such as missiles, nuclear weapons, and fighter jets. As computer control technology has become widely integrated into electromechanical systems, the application of semi-physical simulation has expanded across multiple fields, including aviation, aerospace, automotive, nuclear power, and robotics, garnering broad recognition and playing a pivotal role in the advancement of semi-physical simulation technology [4].

The widespread application of semi-physical simulation technology is evident. Reference [5] developed the EPOS2.0 semi-physical simulation platform for testing the six-degrees-of-freedom dynamics of two satellites in relative motion, contact dynamics during the capture and docking process of the two satellites, sensor-guided methods for measuring space rays and background environment, and guidance. Reference [6] introduced the STVF semi-physical simulation test system, which validated some critical contact collision tasks on SPDM before its space mission. Reference [7] employed a 6-DOF serial robot to simulate the floating base of the operating arm, using a few-degrees-of-freedom robot and motion base on the client side to simulate the motion of client satellites. This simulation is primarily aimed at exploring the application of robotics, visual systems, and automatic control theory in future on-orbit services. Reference [8] designed a semi-physical experimental platform combining robots and air flotation, successfully achieving the free-floating motion of the target during the capture and contact process on a two-dimensional air flotation plane, effectively demonstrating the dynamic motion process of the captured target. Reference [9] addressed safety concerns in industrial robot force/position hybrid control applications and proposed a practical robot collision detection algorithm, verified through semi-physical simulation experiments to achieve effective collision detection.

Reference [10] developed a test system loading platform for ground verification of space robotic arm control algorithms, capable of verifying the stable control effect of space robotic arms when moving heavy loads in vacuum, high- and low-temperature environments, and microgravity conditions. Simulation and experimental results validated the effectiveness of the stable control algorithm for space robotic arms. Reference [11] designed and constructed a semi-physical simulation test platform based on the solid lubrication of mechanical arm joints as the main executive mechanism, integrating physical objects into mathematical simulation and modifying joint contact forces based on experimental results to obtain a joint contact force model suitable for different gravity environments. Reference [12] conducted research on comprehensive, sufficient, and effective testing and verification of space large-scale robotic arms on the ground, proposing a testing and verification system combining semi-physical simulation and physical verification to address the issue of incomplete single-test verification. Reference [13] proposed a fully physical ground simulation test method for space robotic arms, addressing the feasibility issue of on-orbit capture design solutions in a ground environment where space robotic arms cannot support their own weight, thus improving the consistency between ground and orbit experiments. Reference [14] established an experimental platform based on a simulated four-degrees-of-freedom humanoid robotic arm, revealing that the driving torque of the space robotic arm in the ground phase is significantly related to the angular position, whereas in the space phase, the driving torque is less influenced by the angular position. Reference [15] conducted experiments in a ground gravity environment, ground simulated microgravity environment, and drop tower microgravity environment using a single-joint driven robotic arm test device, demonstrating the elimination of gravity influence in the ground environment by aligning the motion axis parallel to the direction of gravity, approximating the space microgravity environment.

This paper addresses the challenges of conducting comprehensive physical experiments in simulated microgravity environments on Earth. It proposes a semi-physical simulation platform for space robotic arms tailored to different gravity environments and loading conditions. The approach involves using physical joints that are difficult to establish accurate mathematical models for and applying equivalent simulations based on the principle of similarity to the parts that are easier to model. The subsequent sections unfold as follows: Section 2 presents the mechanical structure and measurement and control system design of the semi-physical simulation platform for space robotic arms, provides the mathematical model of the electrically variable load system, designs a fractional-order sliding mode controller, and uses particle swarm optimization for parameter tuning. Section 3 presents experimental results and analysis, while Section 4 offers conclusions and suggestions for future work.

## 2. Materials and Methods

The semi-physical simulation platform for space robotic arms is composed of a joint motor attitude control unit, an electric variable load system, and an upper computer UI interface. The joint motor attitude control unit comprises a servo motor and a hollow turntable. The servo motor operates in absolute position mode, enabling the hollow turntable to achieve joint motor attitude control. The electric variable load system includes a radial force loading unit and a load torque loading unit. The radial force loading unit utilizes a servo electric cylinder to extend or retract the front spring mechanism and tension sensor for closed-loop control to implement radial force loading. The load torque loading system employs a torque motor and torque sensor for closed-loop control to simulate load torque loading. Subsequent sections will offer a comprehensive overview of the mechanical structure, operational principles of each component, and the hardware and software design of the semi-physical simulation platform for space robotic arms.

**Figure 1:**
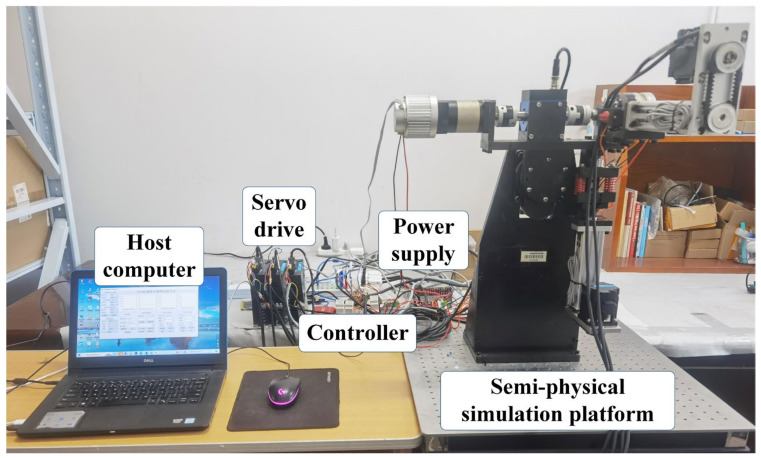
Physical diagram of the semi-physical simulation platform for space robotic arms.

The physical depiction of the space robotic arm semi-physical simulation platform is illustrated in Figure 1, showcasing the complete experimental setup on the right side and the upper computer, controller, power supply, and servo drive on the left side.

### 2.1. Mechanical Structure

**Figure 2:**
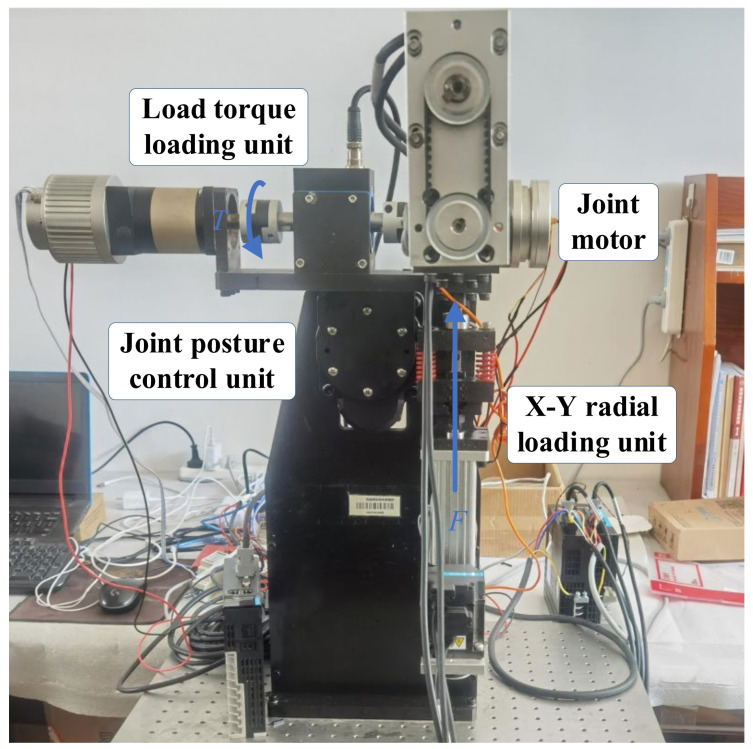
Composition of the mechanical structure.

The semi-physical simulation platform for space robotic arms is designed to treat joints, which poses challenges in establishing precise mathematical models as physical entities. Meanwhile, the easily modelable components are simulated based on the principle of similarity. The equivalent simulation encompasses three main units: the joint attitude control unit, radial force loading unit, and load torque loading unit, as depicted in Figure 2. This simulation platform employs the joint attitude control system to replicate variations in joint attitudes under operational conditions, the radial force loading unit to mimic changes in radial forces acting on the joints, and the load torque loading unit to simulate variations in load torques applied to the joints. It can be utilized for conducting research on joint transmission system testing, joint model identification, refinement of robotic arm dynamics modeling, and analysis of dynamic disparities of robotic arms in diverse environments.

The radial force loading unit comprises servo motors, servo drives, servo electric cylinders, spring mechanisms, and tension sensors. The workpiece on the joint motor shaft serves as the load object for the radial force loading unit. This workpiece contains bearings to mitigate the impact of radial forces on the rotational motion of the joint motor. The radial force is applied perpendicular to the axis of the joint motor. The servo motor operates in absolute position mode and is outfitted with software limits to prevent servo motor overload.

The load torque loading unit consists of a torque motor and torque sensor, connected through a coupling. The joint motor of the space robotic arm on the left side of the figure serves as the load object for the load torque loading unit connected to the torque sensor via a coupling.

The joint attitude control unit is composed of servo motors, servo drives (SV660N, Huichuan Company, Wenzhou, China), and hollow turntables. The servo motor has a rated power of 400 W and features a power-off brake function to ensure the safe operation of the experimental platform. Operating in absolute position mode, the servo motor achieves a control accuracy of up to 0.01°. Through computation, the target joint attitude angles are converted into servo motor position pulse signal numbers, which are then input to the servo drive to control the servo motor in generating corresponding displacements, thereby steering the hollow turntable to achieve joint attitude angle control.

### 2.2. Measurement and Control System

**Figure 3:**
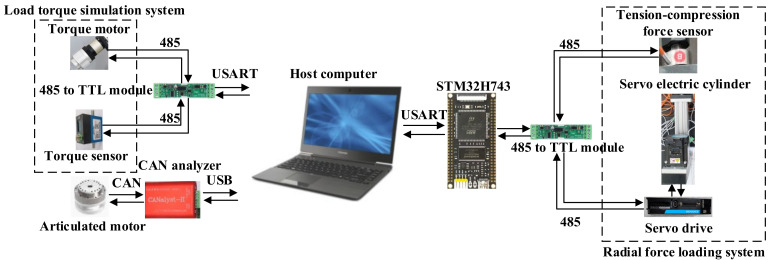
Hardware components of the measurement and control system.

The measurement and control system of the semi-physical simulation platform for space robotic arms is depicted in Figure 3. The upper computer orchestrates the radial force loading unit, load torque loading unit, and joint motor while also recording and storing the current passing through the joint motor, torque sensor, and tension sensor. The upper computer feeds the desired radial force to the controller, which, in turn, regulates the radial force loading unit to apply the corresponding radial force waveform. Subsequently, the microcontroller transmits the tension data back to the upper computer.

**Figure 4:**
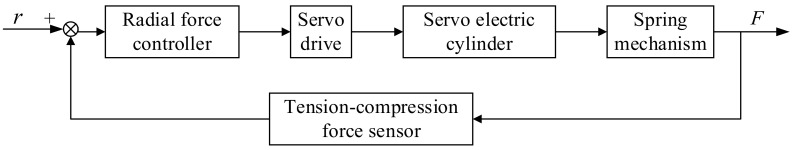
Radial force loading control diagram.

The input to the radial force loading unit is the desired radial force. The controller compares the desired force with the current radial force, sending this deviation to the radial force loading system’s controller. After processing, the controller transmits the control signal, i.e., the position command, to the servo drive (SV660A, Huichuan Company, China). This action prompts the motor to rotate, with the servo electric cylinder transforming the rotary motion into linear motion by stretching or compressing the spring mechanism to generate the appropriate tension. Feedback from the tension sensor (DY500, Dayang Sensing Systems Engineering Co., Ltd., Bengbu, China) at the spring mechanism’s forefront enables closed-loop control of the radial force. The control schematic of the radial force loading unit is outlined in Figure 4.

**Figure 5:**
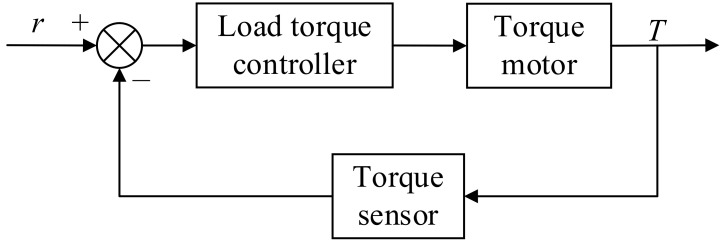
Load torque loading system control diagram.

The operational principle of the load torque loading unit is as follows: by adjusting the input current of the torque motor (60AIM25, Hangzhou Yizhi Technology Co., Ltd., Hangzhou, China), the output torque is modified. This adjustment is made through the alteration of the torque motor’s maximum static output register, with closed-loop control implemented via feedback from the torque sensor (HLT-171, Shenzhen Hualiteng Technology Co., Ltd., Shenzhen, China). As the torque motor functions in speed mode at zero velocity setting, it will be back-driven by the joint motor (LSG-17-70-50, Shanghai Taixin Intelligent Technology Company, Shanghai, China), leading to a power generation state. Accordingly, the experiment duration should be limited to prevent undue strain. The joint motor should operate at low speeds to avert coupling slippage or damage to other components. The control schematic of the load torque loading unit is depicted in Figure 5.

To meet the experimental requisites of the semi-physical simulation platform for space robotic arms, the upper computer’s user interface encompasses sensor data retrieval, joint motor and torque motor control, data uploading and storage, and waveform visualization. The upper computer is primarily divided into sections for communication configuration, variable load management, joint motor orientation setup, joint motor control, and data waveform presentation.

### 2.3. Modeling of Electrically Variable Load Loading System

**Figure 6:**
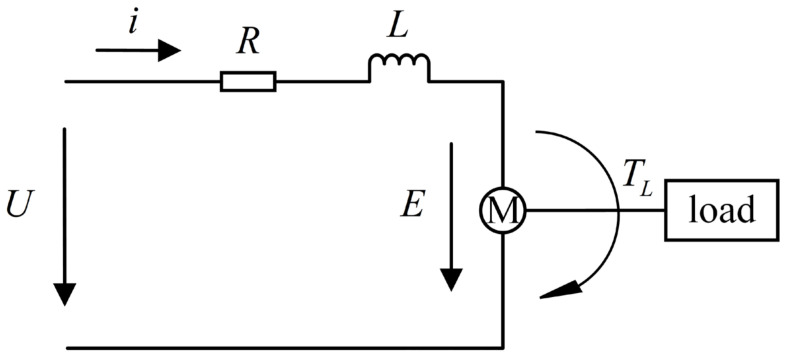
Equivalent circuit diagram of the servo motor.

The equivalent circuit of the servo motor is illustrated in Figure 6.

The voltage balance equation of the servo motor is:

(1) $$U=iR+L\frac{di}{dt}+E$$

The back electromotive force of the motor is:

(2) $$E=K_{e}\omega$$

The torque balance equation of the motor is:

(3) $$T_{m}=J\frac{d\omega }{dt}+B\omega +T_{l}$$

The electromagnetic torque can be expressed as:

(4) $$T_{m}=K_{T}i$$

where U is the armature voltage of the motor; i is the armature current of the motor; R is the total resistance in the armature circuit; E is the back electromotive force of the motor; $K_{e}$ is the back electromotive force coefficient of the motor; ω is the rotational angular velocity of the motor; $T_{m}$ is the electromagnetic torque of the motor; J is the rotational inertia on the motor shaft; B is the damping coefficient of the motor; $T_{l}$ is the output torque on the motor shaft; $K_{T}$ is the torque coefficient of the motor.

By organizing Equations (1)–(4) and performing Laplace transformation, the transfer function of the servo motor model can be obtained as:

(5) $$G_{0}(s)=\frac{\omega (s)}{U(s)}=\frac{K_{T}}{LJs^{2}+(LB+RJ)s+BR+K_{e}K_{T}}$$

The angular displacement of the servo motor is obtained by integrating the angular velocity of the servo motor:

(6) $$\theta (t)=\int_{0}^{t}\omega (t)dt$$

The transmission mechanism of the radial force loading system is a servo electric cylinder, and the linear distance of the output shaft of the servo electric cylinder is:

(7) $$y=\frac{\theta h}{2\pi }$$

Since the deformation of the tension/compression force sensor is small, it is neglected. The tension or compression force output by the spring mechanism is:

(8) $$F=K_{x}y$$

where h is the lead of the ball screw; $K_{x}$ is the spring stiffness coefficient.

Combining Equations (5)–(8), the open-loop transfer function of the experimental loading system is given by:

(9) $$G(s)=\frac{F(s)}{U(s)}=\frac{K_{T}K_{f}h}{2\pi [LJs^{3}+(LB+RJ)s^{2}+(RB+K_{e}K_{T})s]}$$

When the torque motor is uniformly driven, a force analysis of the joint motor yields:

(10) $$T_{l1}=K_{T2}i_{2}+J_{2}\frac{d\omega }{dt}+B_{2}\omega +T_{rf}$$

where $T_{l1}$ is the load of the joint motor; $K_{T2}$ is the torque coefficient of the torque motor; $J_{2}$ is the moment of inertia converted on the torque motor shaft; $B_{2}$ is the damping coefficient of the torque motor; $T_{rf}$ is the excess torque.

**Figure 7:**
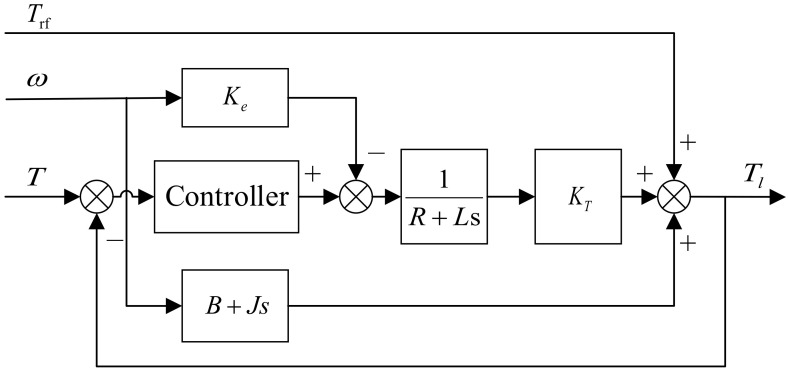
Model diagram of the load torque loading system.

Based on the above equation, the model block diagram of the load torque loading system is depicted in Figure 7. The load on the joint motor, affected by the electromagnetic torque, rotational inertia, friction, and surplus torque of the torque motor, can be controlled by adjusting the output electromagnetic torque of the torque motor and implementing closed-loop control feedback through the torque sensor.

### 2.4. Fractional Order Active Disturbance Rejection Controller Design

**Figure 8:**
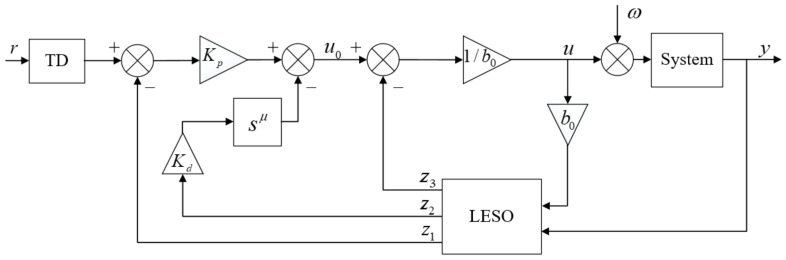
Fractional order active disturbance rejection control principle diagram.

LADRC effectively resolves the contradiction between response speed and overshoot. By employing an extended state observer (ESO) to estimate unmodeled components and external disturbances, it enhances the system’s disturbance rejection performance. To further enhance the control performance of LADRC on the electric variable load loading system, considering the delicacy and information memory characteristics of fractional calculus, along with the increased number of controller parameters to be tuned, the controller’s adjustment range is expanded, thereby improving the system’s dynamic performance [16]. FOLADRC effectively combines the advantages of LADRC and fractional calculus, as depicted in the schematic diagram in Figure 8.

FOLADRC comprises a tracking differentiator (TD), an ESO, and a fractional order proportional-derivative (PD) controller. By integrating the TD and ESO of LADRC, FOLADRC effectively tracks the reference signal, enhances disturbance rejection performance, and introduces fractional calculus to broaden the controller’s adjustment range, increasing its flexibility and improving control effectiveness.

The role of the TD is to process the input signal for smooth transitions, enhancing system speed while reducing overshoot. The expression of the TD is given by:

(11) $$\left\{\begin{array}{l} {\dot{v}}_{1}=v_{2}\\ {\dot{v}}_{2}=-aR^{2}(v_{1}-v_{0})-aRv_{2} \end{array}\right.$$

where R denotes the sampling time, and a is a positive coefficient.

The ESO treats unmodeled components and external disturbances as total disturbances, compensating them into the electric variable load loading system to reduce internal and external disturbances and improve disturbance rejection performance. The expression of the ESO is:

(12) $$\left\{\begin{array}{l} e=z_{1}-y\\ {\dot{z}}_{1}=z_{2}-\beta_{1}e\\ {\dot{z}}_{2}=z_{3}-\beta_{2}e+b_{0}u\\ {\dot{z}}_{3}=-\beta_{3}e \end{array}\right.$$

where $\left[\beta_{1},\beta_{2},\beta_{3}\right]$ is the key parameter of the extended state observer, and appropriate parameter settings can improve the tracking performance of the system. The parameter setting can be carried out by the pole assignment method [17]. According to the engineering experience, the pole can be set as the primary root $\omega_{0}$. $\left[\beta_{1},\beta_{2},\beta_{3}]=[3\omega_{0},3{\omega_{0}}^{2},{\omega_{0}}^{3}\right]$, where $\omega_{0}$ is the bandwidth of the observer; increasing the bandwidth can improve the fast performance and control accuracy of the system, but this results in too large amplification noise, causing system vibration. $b_{0}$ is the output gain.

When the system observation error approaches 0, the state variable estimated by ESO can be approximated as the actual state variable of the system. Therefore, the observer can accurately estimate the state variables of the system.

When fractional calculus is added to linear combination, the digital realization of fractional differential is infinite dimensional, which needs to be approximated by finite dimensional differential equation in a certain frequency range. The time domain approximation of fractional calculus operator obtained according to G-L definition [18] is:

(13) Dtβt0f(t)≈Dtβt−Lf(t)≈h−β∑j=0M(t)wjβf(t−jh)

where L is the memory length; $M(t)=\min\{[\frac{t}{h}][\frac{L}{h}]\}$, where smaller h and larger L means the approximate calculus effect is more accurate.

The state error feedback of FOLADRC is transformed into a discrete time series equation and obtained as:

(14) $$u_{0}(k)=K_{p}e_{1}(k)+K_{d}e_{2}(k)$$

where the discretization expressions for $e_{1}$ and $e_{2}$ are as follows:

(15) $$\left\{\begin{array}{l} e_{1}(k)=x_{1}(k)-z_{1}(k)\\ e_{2}(k)=-h^{-\mu }\sum_{j=0}^{k}w_{j}^{\mu }z_{2}(k-j) \end{array}\right.$$

where j=1,2…,k, ω is the weight coefficient, and the derivation formula is as follows:

(16) $$\omega_{0}^{\mu }=1,\omega_{j}^{\mu }=(1-\frac{1+\mu }{j})\omega_{j-1}^{\mu }$$

In addition to the two parameters of TD, FOLADRC requires the tuning of five additional parameters. The tuning of controller parameters is challenging, and only by selecting appropriate parameters can the desired control effect be achieved. Therefore, the particle swarm optimization algorithm is employed for controller parameter tuning.

The particle swarm optimization algorithm, proposed by Eberhart and Kennedy, simulates the way birds seek food during flight and is a type of swarm intelligence optimization algorithm. Nowadays, the particle swarm algorithm has been widely applied in various research endeavors [19].

The basic principle of particle swarm optimization [20]: suppose a D-dimensional space is taken as the target search space, N particles are taken as a particle swarm population in the space; suppose the position and velocity of the i particle are $x_{i}=\left(x_{i1},x_{i2},\cdot \cdot \cdot ,x_{iD}\right)$ and $v_{i}=\left(v_{i1},v_{i2},\cdot \cdot \cdot ,v_{iD}\right)$, respectively, and the optimal position sought by this particle in the whole target search process is recorded as the individual extreme value $p_{\mathrm{best}}=\left(p_{i1},p_{i2},\cdot \cdot \cdot ,p_{iD}\right)$ of this particle. The optimal position sought by the whole N particle population in the process of target search is recorded as the global extreme value $g_{\mathrm{best}}=\left(p_{g1},p_{g2},\cdot \cdot \cdot ,p_{gD}\right)$ of the whole particle population. In the iteration process, the particle updates its speed and position according to the individual extreme value and global extreme value until the iteration termination condition is met. The speed and position iteration formula of the particle swarm algorithm is as follows:

(17) $$\left\{\begin{array}{l} v_{id}^{k+1}=\omega v_{id}^{k}+c_{1}r_{1}(P_{id}^{k}-x_{id}^{k})+c_{2}r_{2}(p_{gd}^{k}-x_{id}^{k})\\ x_{id}^{k+1}=x_{id}^{k}+v_{id}^{k+1} \end{array}\right.$$

where *i* (*i* = 1, 2, …, *N*) is the population number; *d* (*d* = 1, 2, …, D) is the spatial dimension; *k* is the current number of iterations; $c_{1}$ and $c_{2}$ are weight coefficients; $r_{1}$ and $r_{2}$ are both random numbers distributed in the interval [0, 1]; ω is the inertia weight, which determines the global and local search capability of the algorithm. When the inertia weight is larger, the global search ability of the particle swarm is improved; when the particle swarm is smaller, the local search ability of the particle swarm is improved. Usually, $\omega_{\max}=0.9$, $\omega_{\min}=0.4$; when the weight coefficient is taken, the algorithm has the best performance, the ability to find the global optimal value in a reasonable number of iterations is better, and the number of iterations required is the least [21]. At present, the commonly used inertia weight formula is:

(18) $$\omega =\omega_{\max}-\frac{(\omega_{\max}-\omega_{\min})t}{t_{\max}}$$

where $t_{\max}$ is the maximum number of iterations; t is the current iteration times.

Although ω is no longer a fixed value through this formula, the global search ability is enhanced in the early stage of iteration, and the local search ability is enhanced in the later stage, but the change rate of ω is a fixed value, and it may not be better to seek the optimal value under strong search conditions.

In practical applications, there are many uncertainties. The change rate of ω in the above formula is a fixed value, and the optimal value may not be obtained under strong search conditions. Inspired by the literature [22], this paper introduces the 1/4 period of sine function and improves the weight factor, making w as follows:

(19) $$\left\{\begin{array}{l} a=t/t_{\max}\\ b=\sin[\pi /2\cdot (a-1)]+1\\ \omega =\omega_{\max}\cdot (1-b^{3})+\omega_{\min}\cdot rand\cdot b^{3} \end{array}\right.$$

The improvement of ω introduces stochastic function to increase randomness in particle swarm iteration. ω changes slowly in the early stage of particle swarm iteration and takes a large value, so the global search ability of particle swarm is strong. In the later stage, the change is fast, the value is small, and the local search ability of PSO is strong, which is conducive to preventing PSO from falling into the local optimal and obtaining the controller parameters with better control effect.

ITAE integral evaluation function was used as fitness function:

(20) $$ITAE=\int_{0}^{\infty }t\left|e(t)\right|dt$$

where e(t) is the error.

When PSO is used to adjust the linear active disturbance rejection parameters, the large fitness selection is conducive to reducing system overshoot, but it will reduce the fast performance. Small fitness selection is conducive to improving the fast performance of the system, but it will increase the overshoot of the system and even produce shock, so the appropriate fitness should be selected.

**Figure 9:**
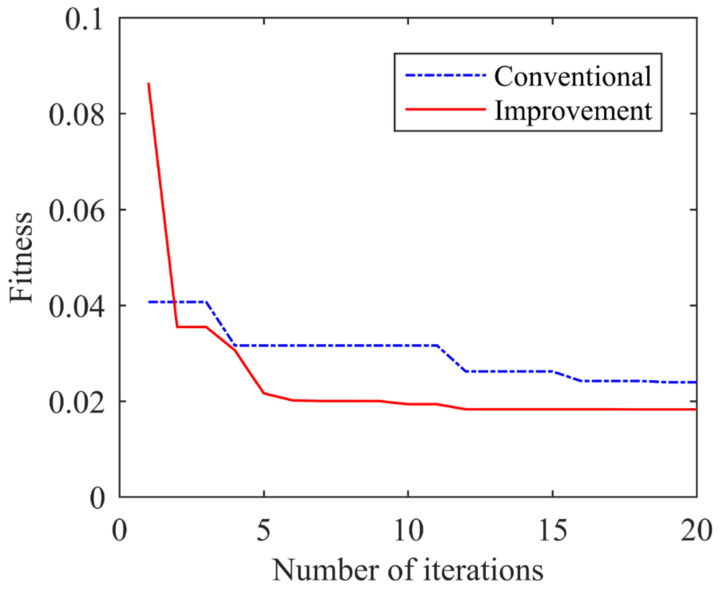
Adaptation value variation curve.

Conventional ω and improved ω were used to adjust FOLADRC controller parameters, respectively. The change curves of their adaptation values are shown in Figure 9. It can be seen that improved ω tuning parameters can find better controller parameters faster.

**Figure 10:**
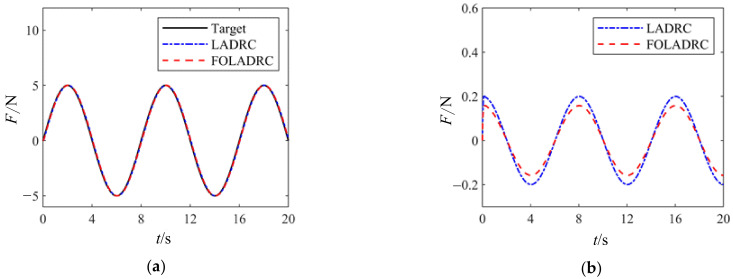
Particle swarm algorithm optimized sine wave tracking curve. (**a**) Tracking curve; (**b**) error curve.

In order to explore the influence of the improved ω particle swarm optimization algorithm on this experiment, the parameters adjusted by the improved ω and the conventional ω were simulated. The tracking and error curves of sine wave signal load with input pressure load amplitude of 5 N and period of 8 s are shown in Figure 10.

It can be seen from Figure 10a that both kinds of ω optimization have obtained better tracking effects, and from the error curve in Figure 10b, it can be seen that the error after the optimization with improved ω is small, especially the error near the zero point of the sine wave.

## 3. Experiments

To investigate the control performance of FOLADRC in practical systems, experiments were conducted on an electric variable load loading system under both stationary and rotating joint motor conditions. Real-time data from tension and compression force sensors were collected by the host computer, and waveform plots were generated. Constant, square wave, triangular wave, and sinusoidal waveforms were used as input signals for variable load loading experiments. Real-time storage of experimental data was performed, and corresponding load tracking curves and error curves were plotted based on the stored data. During dynamic loading, the motor speed was set to 50 r/min, simulating the introduction of external disturbances when the joint motor rotates.

**Figure 11:**
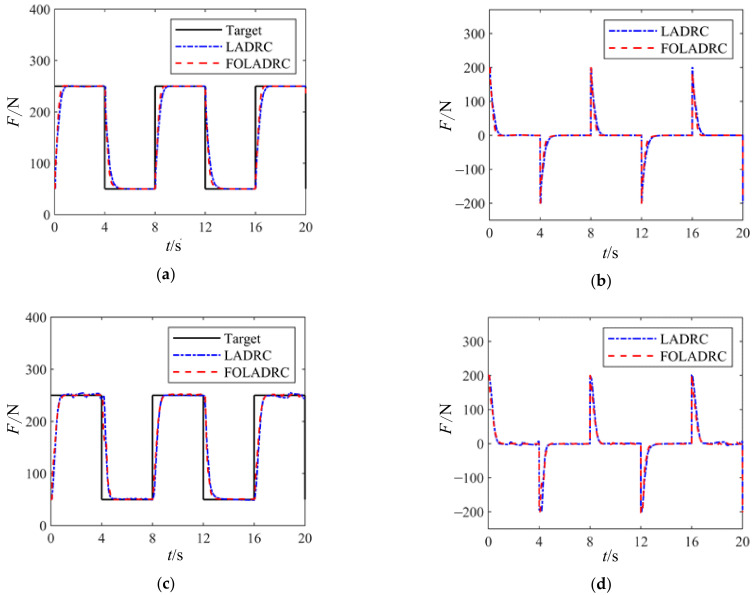
Square wave loading experiment curve. (**a**) Joint motor tracking curve when stationary. (**b**) Error curve of joint motor when stationary. (**c**) Joint motor tracking curve during rotation. (**d**) Error curve of joint motor during rotation.

Figure 11 shows the load tracking curves and error curves of the electric variable load loading system under a square wave signal with a peak load of 250 N, a valley load of 50 N, and an 8-s period. Panels (a) and (b) represent the system without external disturbance, while panels (c) and (d) depict the system with external disturbance introduced during joint motor rotation.

From Figure 11, it can be observed that without external disturbances, both methods exhibit good control performance, achieving no overshoot. FOLADRC demonstrates a faster response speed, with a reduction in rise time of approximately 0.12 s. When external disturbances are introduced, the response speeds of both methods are similar, but FOLADRC exhibits smaller oscillations after stabilization, indicating stronger disturbance rejection capability.

**Figure 12:**
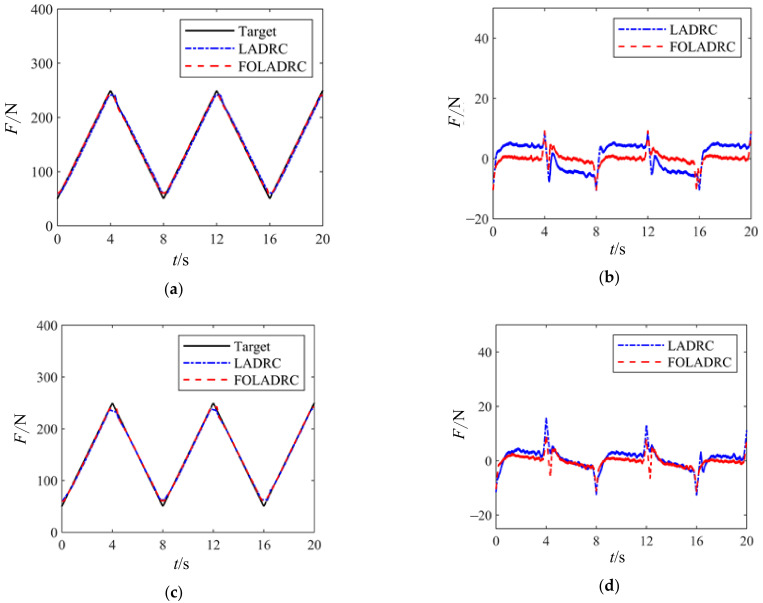
Triangle wave loading experiment curve. (**a**) Joint motor tracking curve when stationary. (**b**) Error curve of joint motor when stationary. (**c**) Joint motor tracking curve during rotation. (**d**) Error curve of joint motor during rotation.

Figure 12 shows the load tracking curves and error curves of the electric variable load loading system under a triangular wave signal with a peak load of 250 N, a valley load of 50 N, and an 8-s period. Panels (a) and (b) represent the system without external disturbance, while panels (c) and (d) depict the system with external disturbance introduced.

From Figure 12, it can be observed that in the absence of external disturbance, the tracking error of the electric variable load loading system is smaller with FOLADRC compared to LADRC, and both show some error at the peaks. When an external disturbance is present in the system, FOLADRC exhibits smaller tracking errors compared to LADRC but displays oscillations.

The data analysis from the above simulations and experiments indicates that FOLADRC, by introducing a fractional-order differential factor compared to LADRC, reduces overshoot, enhances system disturbance rejection capabilities, and improves response speed.

Loading experiments were conducted on the load torque loading system of a semi-physical simulation platform for space manipulator arms, employing a constant waveform. Real-time data acquisition was utilized to capture and store experimental data, enabling the generation of corresponding tracking and error curves based on the stored experimental data.

**Figure 13:**
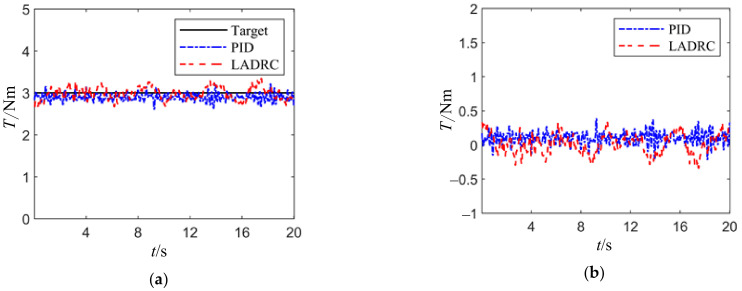
Constant load experiment curve. (**a**) Tracking curve; (**b**) error curve.

Figure 13 illustrates the load tracking curves and error curves of the load torque loading system under a constant signal load of 3 Nm, controlled by traditional PID and LADRC methods.

From the figure, it is evident that both LADRC and PID exhibit commendable tracking performance when following constant value signals. The PID controller demonstrates a smaller tracking error compared to LADRC, highlighting its superior performance in practical applications, especially in disturbance rejection. Consequently, PID control will be implemented for regulating the loading torque in the upcoming experiments.

In order to investigate the effects of different gravity environments and loading methods on the drive current of joint motors, the control algorithm discussed above was applied to the electric variable load loading system. Joint motor drive force experiments were conducted through the upper computer UI interface. Detailed experimental descriptions will follow.

**Figure 14:**
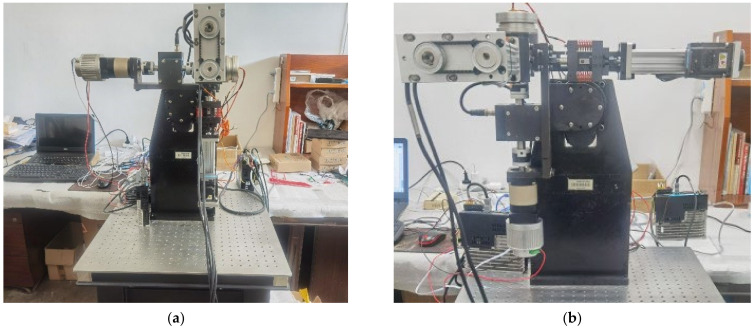
Experimental platform with different joint posture angles. (**a**) The posture angle is 0° (gravity environment). (**b**) The posture angle is 90° (simulated microgravity environment).

The joint posture angle is set to 0° when the joint motor axis is parallel to the horizontal plane, representing the ground gravity environment, and 90° when the joint motor axis is perpendicular to the horizontal plane, representing the ground simulated microgravity environment, as shown in Figure 14.

**Figure 15:**
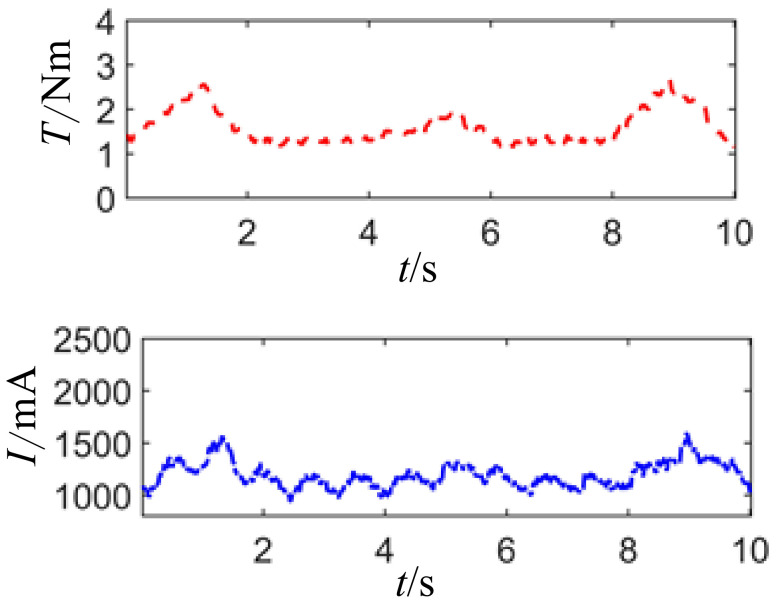
Current waveform diagram of unloaded joint motor drive.

Figure 15 illustrates the curves of joint motor drive current and load torque under a joint posture of 0° and no-load condition. The joint motor drive current fluctuates with the variation of load torque, with the fluctuation being attributed to the unstable speed of the joint motor. Meanwhile, the fluctuation in joint motor drive current is caused by the instability of the load, which is primarily due to the influence of excess torque and the unstable speed of the joint motor.

**Figure 16:**
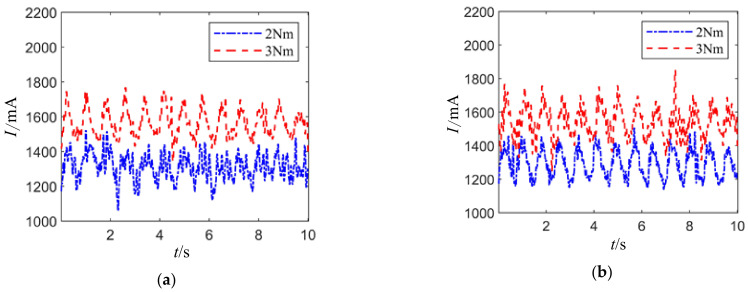
Different load joint motor drive current waveforms. (**a**) The attitude angle is 0°. (**b**) The attitude angle is 90°.

With the joint motor posture angles at 0° and 90°, different loads of torque are applied axially. When there is no radial load, the joint motor drive current fluctuates, as shown in Figure 16. It can be observed that the larger the axial load torque, the larger the drive current, indicating that the load torque is the main factor affecting the joint motor drive current. The difference between (a) and (b) shows that the drive current at a joint angle of 90° is relatively smaller than at 0°, but the data comparison is not significant enough and will be further described.

**Figure 17:**
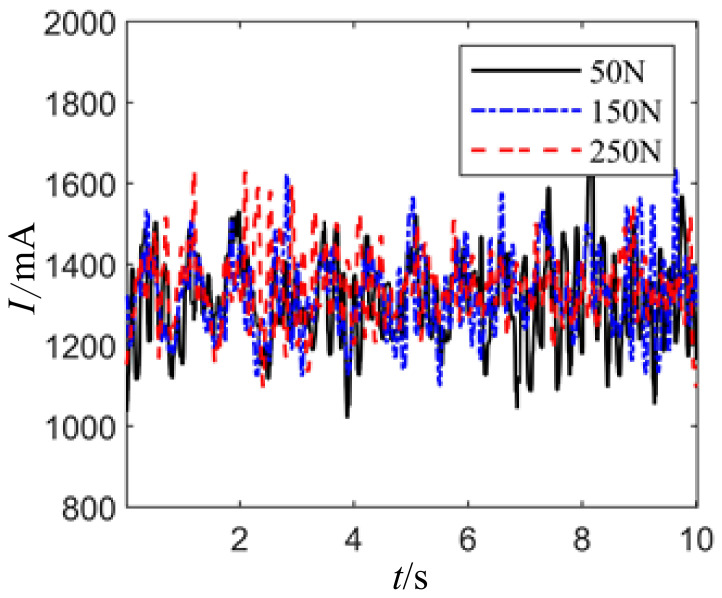
Under constant value waveform loading with different amplitudes, the joint motor drive current waveforms.

When the joint motor posture angle is set to 0°, meaning the joint motor axis is parallel to the ground, with axial load being zero (static output of torque motor is maximum), and applying constant and peak values of 250 N and valley values of 50 N with 8-s period square wave, triangular wave, and sine wave radial forces, the resulting joint motor drive current waveform is shown in Figure 17.

**Figure 18:**
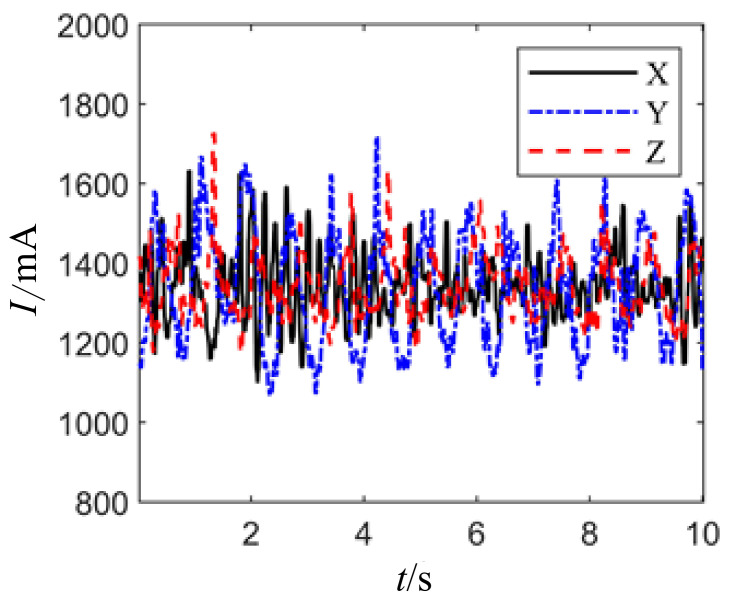
The waveform of joint motor drive current when loading constant waveforms in different radial directions.

When the load torque is 2 Nm, the resultant force direction generated by equally loaded radial forces in the X and Y directions is in the Z direction. Radial forces of 250 N each are loaded in the X and Y directions, with 250 N loaded in the Z direction achieved through loading 176.8 N in the X and Y directions, respectively. The current waveform of the joint motor is shown in Figure 18, with average driving currents of 1339.7 mA, 1335.3 mA, and 1335.9 mA for the joint motor. It is illustrated that when the same magnitude of load torque and radial force are applied, the driving current of the joint motor is essentially independent of the direction of the radial force.

**Figure 19:**
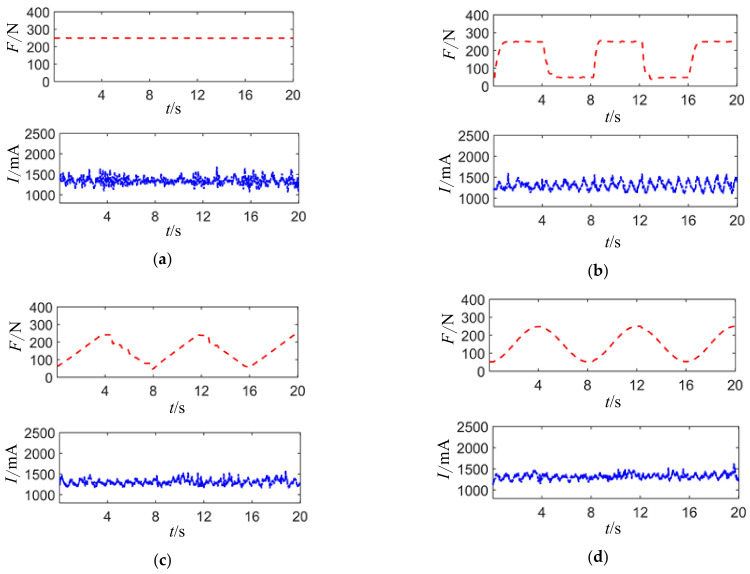
Current waveforms of joint motor drive under different radial force loadings. (**a**) Constant load and driving current. (**b**) Square wave load and driving current. (**c**) Triangle wave load and driving current. (**d**) Sine wave load and driving current.

Setting the joint motor posture angle to 0°, meaning the axis of the joint motor is parallel to the ground, with axial load unloaded (maximum static output torque of the motor is 0), a constant radial force of 250 N, a peak value of 250 N, a valley value of 50 N, and periodic square wave, triangular wave, and sinusoidal wave radial forces with a period of 8 s, the waveform of the joint motor driving current is shown in Figure 19.

It can be observed that the joint motor driving current exhibits similar trends with the variations in the radial force waveform. However, the fluctuation in the joint motor driving current caused by the radial force fluctuation is relatively smaller compared to the fluctuation caused by the load torque, further proving that the radial force is a secondary influencing factor on the joint motor driving current.

It can be seen that the joint motor drive current fluctuates in the same trend as the radial force waveform changes, but the fluctuation in the drive current caused by radial force is relatively smaller compared to that caused by load torque fluctuation, further confirming that radial force is a secondary factor affecting the joint motor drive current.

**Figure 20:**
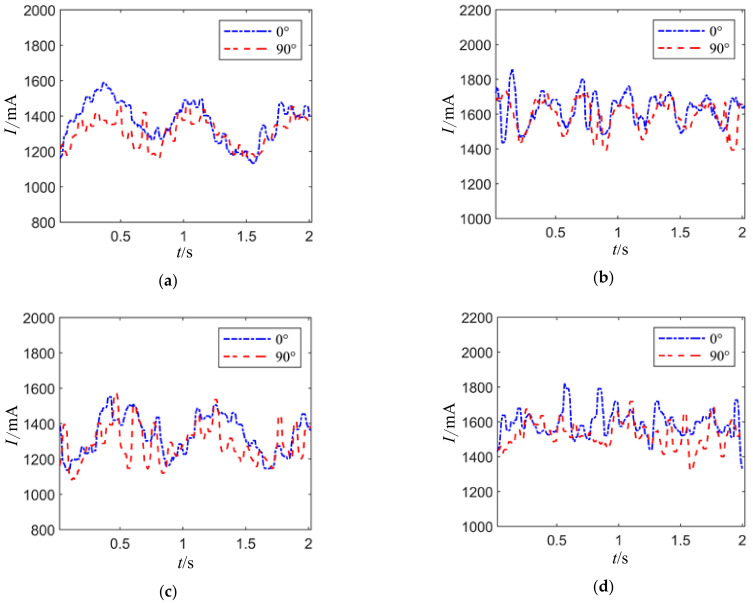
Joint motor drive current waveforms at different joint pose angles. (**a**) The load torque is 2 Nm with radial free load. (**b**) The load torque is 3 Nm with radial free load. (**c**) The load torque is 2 Nm with a radial force of 250 N. (**d**) The load torque is 3 Nm with a radial force of 250 N.

The current waveform of the joint motor under different joint posture angles is shown in Figure 20, where (a) and (b) represent the motor drive current waveform with axial loads of 2 Nm and 3 Nm, respectively, and no radial load at different joint motor postures. In (a), the average motor drive current at a joint posture angle of 0° is 1306.8 mA, and at 90°, it is 1289.7 mA; in (b), the average motor drive current at a joint posture angle of 0° is 1558.3 mA, and at 90°, it is 1534.9 mA. It indicates that without radial load, when the same load torque is applied, the joint motor drive current in the simulated ground microgravity environment is smaller compared to the gravity environment.

Figure 20c,d show the motor drive current waveform with axial loads of 2 Nm and 3 Nm, respectively, and a radial force of 250 N. In (c), the average motor drive current at a joint posture angle of 0° is 1343.2 mA, and at 90°, it is 1298.8 mA; in (d), the average motor drive current at a joint posture angle of 0° is 1568.0 mA, and at 90°, it is 1547.3 mA. It can be observed that when the same load is applied, the joint motor drive current at a posture angle of 90° is smaller compared to 0°, indicating that in the simulated ground microgravity environment, the motor drive current is lower than in the gravity environment.

## 4. Conclusions

This study introduces the development of a semi-physical simulation platform tailored for robotic arms in various gravity environments and loading conditions. It elaborates on the mechanical structure, design of measurement and control systems, as well as the operational principles of the experimental platform. To tackle issues like strong coupling, nonlinearity, and excessive force disturbance in the electric variable load loading system, fractional calculus is incorporated into LADRC to derive FOLADRC. The parameters of the FOLADRC controller are fine-tuned using an enhanced particle swarm algorithm with adjusted weight coefficients. Results demonstrate that compared to LADRC, FOLADRC reduces the static rise time by approximately 0.12 s and displays reduced susceptibility to external disturbances such as joint motor rotation during dynamic tracking, thereby significantly minimizing errors and improving disturbance rejection performance. The research explores the impact of diverse gravity environments and loading conditions on the drive force of joint motors in space robotic arms. Experimental findings reveal that with increasing load torque, the drive force of joint motors also increases, with load torque being the primary influencing factor on joint motor drive force. Variations in drive force occur when different waveforms of radial force are applied. Radial force alters joint motor drive force by modifying the friction torque of the joint motor, acting as a secondary influencing factor. Radial forces of equivalent magnitude but different directions consistently affect joint motor drive force. Furthermore, under identical loading conditions, when the axis of the joint motor is perpendicular to the ground, the drive force of the joint motor relative to the gravity environment diminishes, thereby partially emulating microgravity conditions on Earth.

To address the subpar tracking performance of triangular wave signals by the electric variable load loading system, forward compensation methods will be integrated into the current solution to enhance the tracking accuracy of triangular wave peaks. Furthermore, future efforts will involve comparative analysis utilizing different control schemes.

## Data Availability

The authors reserve the right not to disclose the private dataset used in this study.
